# Multiple Hits in Acute Pancreatitis: Components of Metabolic Syndrome Synergize Each Other’s Deteriorating Effects

**DOI:** 10.3389/fphys.2019.01202

**Published:** 2019-09-20

**Authors:** Andrea Szentesi, Andrea Párniczky, Áron Vincze, Judit Bajor, Szilárd Gódi, Patricia Sarlós, Noémi Gede, Ferenc Izbéki, Adrienn Halász, Katalin Márta, Dalma Dobszai, Imola Török, Hunor Farkas, Mária Papp, Márta Varga, József Hamvas, János Novák, Artautas Mickevicius, Elena Ramirez Maldonado, Ville Sallinen, Dóra Illés, Balázs Kui, Bálint Erőss, László Czakó, Tamás Takács, Péter Hegyi

**Affiliations:** ^1^Institute for Translational Medicine, Szentágothai Research Centre, Medical School, University of Pécs, Pécs, Hungary; ^2^First Department of Medicine, University of Szeged, Szeged, Hungary; ^3^Doctoral School of Clinical Medicine, University of Szeged, Szeged, Hungary; ^4^Heim Pál National Institute of Pediatrics, Budapest, Hungary; ^5^Division of Gastroenterology, First Department of Medicine, Medical School, University of Pécs, Pécs, Hungary; ^6^Division of Translational Medicine, First Department of Medicine, Medical School, University of Pécs, Pécs, Hungary; ^7^Szent György Teaching Hospital of Fejér County, Székesfehérvár, Hungary; ^8^County Emergency Clinical Hospital – Gastroenterology and University of Medicine, Pharmacy, Sciences and Technology, Târgu Mureş, Romania; ^9^Division of Gastroenterology, Department of Internal Medicine, University of Debrecen, Debrecen, Hungary; ^10^Dr. Réthy Pál Hospital of Békés County, Békéscsaba, Hungary; ^11^Bajcsy-Zsilinszky Hospital, Budapest, Hungary; ^12^Department of Gastroenterology, Pándy Kálmán Hospital of Békés County, Gyula, Hungary; ^13^Vilnius University Hospital Santaros Clinics, Vilnius, Lithuania; ^14^Clinics of Abdominal Surgery, Nephrourology and Gastroenterology, Faculty of Medicine, Vilnius University, Vilnius, Lithuania; ^15^Consorci Sanitari del Garraf, Sant Pere de Ribes, Barcelona, Spain; ^16^Department of Transplantation and Liver Surgery, Helsinki University Hospital and University of Helsinki, Helsinki, Finland; ^17^Hungarian Academy of Sciences – University of Szeged, Momentum Gastroenterology Multidisciplinary Research Group, Szeged, Hungary

**Keywords:** acute pancreatitis, metabolic syndrome, obesity, diabetes mellitus, hypertension, hyperlipidemia, severity, mortality

## Abstract

**Introduction:**

The incidence of acute pancreatitis (AP) and the prevalence of metabolic syndrome (MetS) are growing worldwide. Several studies have confirmed that obesity (OB), hyperlipidemia (HL), or diabetes mellitus (DM) can increase severity, mortality, and complications in AP. However, there is no comprehensive information on the independent or joint effect of MetS components on the outcome of AP. Our aims were (1) to understand whether the components of MetS have an independent effect on the outcome of AP and (2) to examine the joint effect of their combinations.

**Methods:**

From 2012 to 2017, 1435 AP cases from 28 centers were included in the prospective AP Registry. Patient groups were formed retrospectively based on the presence of OB, HL, DM, and hypertension (HT). The primary endpoints were mortality, severity, complications of AP, and length of hospital stay. Odds ratio (OR) with 95% confidence intervals (CIs) were calculated.

**Results:**

1257 patients (55.7 ± 17.0 years) were included in the analysis. The presence of OB was an independent predictive factor for renal failure [OR: 2.98 (CI: 1.33–6.66)] and obese patients spent a longer time in hospital compared to non-obese patients (12.1 vs. 10.4 days, *p* = 0.008). HT increased the risk of severe AP [OR: 3.41 (CI: 1.39–8.37)], renal failure [OR: 7.46 (CI: 1.61–34.49)], and the length of hospitalization (11.8 vs. 10.5 days, *p* = 0.020). HL increased the risk of local complications [OR: 1.51 (CI: 1.10–2.07)], renal failure [OR: 6.4 (CI: 1.93–21.17)], and the incidence of newly diagnosed DM [OR: 2.55 (CI: 1.26–5.19)]. No relation was found between the presence of DM and the outcome of AP. 906 cases (mean age ± *SD*: 56.9 ± 16.7 years) had data on all four components of MetS available. The presence of two, three, or four MetS factors increased the incidence of an unfavorable outcome compared to patients with no MetS factors.

**Conclusion:**

OB, HT, and HL are independent risk factors for a number of complications. HT is an independent risk factor for severity as well. Components of MetS strongly synergize each other’s detrimental effect. It is important to search for and follow up on the components of MetS in AP.

## Introduction

Acute pancreatitis is a severe inflammatory condition with increasing incidence and hospitalization worldwide (Forsmark et al., 2016; Garg et al., 2019). AP has a variable severity ranging from mild and self-limited to severe and fatal. The mortality of the disease ranges approximately from 2 to 5% and depends on the development of organ failure and local complications, which are summarized in the revised Atlanta classification (Banks et al., 2013). The major etiological factors are gallstones and alcohol consumption (Forsmark et al., 2016), but hypertriglyceridemia (HTG) and intake of certain medications may also be in the background.

The severity and outcome of AP are influenced by the metabolic comorbidities of the host (Working Group Iap/Apa Acute Pancreatitis Guidelines, 2013; Goodger et al., 2016). Metabolic syndrome is characterized by the clustering of abdominal OB, HTG, low levels of high-density lipoprotein (HDL), elevations in blood pressure and fasting glucose, or diabetes (Alberti et al., 2009). MetS is associated with an increased risk of development of and death from cardiovascular disease and chronic kidney disease (Isomaa et al., 2001). The presence of MetS was previously shown to be associated with a higher risk of severe AP, higher mortality rate, and longer duration of stay in the intensive care unit (Mikolasevic et al., 2016). However, in another study, MetS did not affect the severity of AP (Sawalhi et al., 2014). OB was previously shown to be independently associated with the severity of AP (Sawalhi et al., 2014) and the development of organ failure but not with mortality in AP (Smeets et al., 2019). DM was associated with a higher risk of AP (Yang et al., 2013) and negatively influenced the outcome of AP by raising the incidence of renal failure, intensive care unit admission, and length of hospital stay (LOS) (Miko et al., 2018). The presence of HTG increased severity, complication rate, and mortality in AP (Kiss et al., 2018).

However, there is no data regarding a link between the outcome of AP and the presence of arterial HT. Furthermore, there is a lack of data on how the components of MetS, namely, OB, DM, HT, and HL, influence the outcome of AP individually or in combination. Therefore, in this study, we aimed to analyze how the components of MetS influence the outcome of AP (1) individually and (2) in combination.

## Materials and Methods

### Patient Population and Study Design

The APR launched in 2011 by the Hungarian Pancreatic Study Group is an international prospective registry for patients suffering from AP. Besides pancreatic registries, HPSG has already organized five registered clinical trials to investigate AP with the acronyms PREPAST (Dubravcsik et al., 2015), APPLE (Parniczky et al., 2016), PINEAPPLE (Zsoldos et al., 2016), GOULASH (Marta et al., 2017), and EASY (Hritz and Hegyi, 2015) and has submitted three further pre-study protocols: GOULASH PLUS (follow-up to the GOULASH study), EMILY (endoscopic sphincterotomy for delaying cholecystectomy in mild acute biliary pancreatitis), and LIFESPAN (lifestyle, prevention, and risk of AP).

From June 2012 to September 2017, 1435 adult patients with AP from 28 community and university hospitals were prospectively enrolled (Supplementary Appendix S1). Demographic and anthropometric data; history of HL, HT, and DM; previous medical therapy and etiology; severity; local and systemic complications; and mortality of AP were collected.

In this study, we aimed to maximize the number of cases for each individual effect analysis. We had information concerning OB from 1257 cases, HT from 1127 cases, DM from 1257 cases, and HL from 1036 cases. Patients were grouped based on the World Health Organization (WHO) classification of BMI (≥30 or <30 kg/m^2^) and the presence or absence of three other components, HT, HL, and DM. However, in the “joint effect analysis,” we only included cases where data from all four components of MetS, OB, HL, HT, and DM were available (906 cases). We conducted an additional analysis to confirm that the cohorts noted above represent the total cohort of 1435 cases. Importantly, there were no significant differences in demographics or the main outcome parameters between the cohorts (Supplementary Appendix S2).

Data were collected by treating physicians with the help of trained and experienced study administrators on the basis of a standardized case report form and protocol in the prospective APR. Accuracy of data recorded is secured by a four-level quality check system involving both medical administrative personnel and gastroenterologists. Data quality is presented in Supplementary Appendix S3. The study protocol was approved by the Scientific and Research Ethics Committee of the Medical Research Council (22254-1/2012/EKU). All patients provided written informed consent to participate in the registry.

### Definitions

Diagnosis of AP was made according to the recommendations in the IAP/APA guidelines. At least two criteria of the following three were present: upper abdominal pain, pancreatic enzyme levels exceeding more than three times the upper normal level, and features of pancreatitis on imaging (Working Group Iap/Apa Acute Pancreatitis Guidelines, 2013). Severity and complications of AP were determined according to the revised Atlanta classification (Banks et al., 2013). OB was determined if BMI was ≥30 kg/m^2^ (Jensen et al., 2014). HT was determined if blood pressure was ≥140/90 mmHg or if the patient was on anti-hypertensive medication. HL was defined by the presence of either hypercholesterolemia or a low level of HDL or HTG. The condition was regarded as HL when fasting cholesterol level >200 mg/dL (5.2 mmol/L), HDL < 44 mg/dL (1.15 mmol/L; female) or <35 mg/dL (0.9 mmol/L; male), triglyceride level exceeded 150 mg/dL (1.7 mmol/L), or the patient was receiving drug therapy for HL. The diagnosis of DM was made in accordance with the American Diabetes Association Criteria (American Diabetes Association, 2010) or if the patient was receiving drug therapy for hyperglycemia.

The primary endpoints were mortality, severity, and complications of AP and LOS.

### Statistical Analyses

Case numbers and percentages were calculated for categorical variables, mean with *SD*, and medians with 25 and 75% quartiles (Q1 and Q3, respectively) and ranges were computed for numerical variables in descriptive analysis.

The *t*-test was used for normally distributed data and the Mann–Whitney *U*-test for non-normally distributed data to compare two groups of independent samples. The relation between categorical variables was inspected by the Chi-square test and *Z*-test with the Bonferroni correction and ORs with 95% CIs.

Logistic regression was used to define the independent effect of the MetS factors and age. A two-sided *p*-value of <0.05 was regarded as statistically significant. The available-case analysis was used for missing data. Statistical analyses were performed with SPSS 25.0 software (IBM Corporation).

## Results

### Individual Effect Analysis

A total of 1257 patients (mean age ± *SD*: 55.7 ± 17.0 years, males vs. females: 57.1 vs. 42.9%) were recruited for the “individual effect analysis.” 371 patients (29.5%) had OB, 676 (60.0%) had HT, 349 (33.7%) had HL, and 206 (16.4%) had DM (Table 1).

**TABLE 1:** Individual effect analysis.

	**Total cohort**	**Obesity (*n* = 1257)**		**Hypertension (*n* = 1127)**		**Hyperlipidemia (*n* = 1036)**		**Diabetes mellitus (*n* = 1257)**	
		**Non-OB**	**OB**	**Non-HT**	**HT**	**Non-HL**	**HL**	**Non-DM**	**DM**
*n*	1257	886	371	451	676	687	349	1051	206
% within groups		70.5	29.5	40.0	60.0	66.3	33.7	83.6	16.4
**Age, sex, CCI**									
Average age	55.7	55.4	56.3	**46.2**	**63.8^∗^**	**56.4**	**54.0^∗^**	**54.5**	**61.7^∗^**
*SD* (average age)	17.0	17.7	15.2	15.2	14.1	17.8	14.5	17.3	13.9
Male (%)	57.1	59.3	52.0	61.9	51.8	**55.6**	**64.8^∗^**	56.4	60.7
Female (%)	42.9	**40.7**	**48.0^∗^**	**38.1**	**48.2^∗^**	44.4	35.2	43.6	39.3
Average CCI	1.4	1.3	1.6	0.9	1.7	1.3	1.7	1.0	2.9
*SD* (CCI)	1.6	1.6	1.7	1.4	1.7	1.6	1.8	1.4	1.7
**Etiology (%)**									
Biliary	37.8	**33.6**	**47.7^∗^**	31.3	44.1	41.3	26.4	38.2	35.9
Alcoholic	18.5	21.1	12.1	20.2	12.4	21.4	17.2	19.0	15.5
HTG-induced	3.7	3.0	5.4	3.3	3.7	**0.1**	**12.9^∗^**	**2.8**	**8.7^∗^**
Alcoholic + HTG-induced	1.8	1.9	1.6	1.6	1.9	0.0	6.6	1.8	1.9
Post-ERCP	2.6	3.0	1.6	3.1	2.8	2.9	0.9	2.6	2.9
Combined	8.0	7.1	10.0	11.1	7.0	7.7	7.2	7.9	8.3
Idiopathic	20.5	22.0	17.0	21.5	20.7	18.8	23.8	20.6	20.4
Other	7.1	8.1	4.6	8.0	7.4	7.7	5.2	7.2	6.3
**Severity, mortality, LOS**									
Mild (%)	69.6	69.9	69.0	70.1	69.5	**73.5**	**64.2^∗^**	69.7	68.9
Moderate (%)	25.1	26.1	22.6	26.8	23.4	22.1	29.5	24.9	25.7
Severe (%)	5.3	**4.1**	**8.4^∗^**	**3.1**	**7.1^∗^**	4.4	6.3	5.3	5.3
Mortality (%)	2.4	2.1	3.0	1.3	3.1	2.3	1.4	2.5	1.9
Average LOS	10.9	**10.4**	**12.1^∗^**	**10.5**	**11.8^∗^**	10.5	11.4	10.7	11.8
*SD* (LOS)	9.3	8.6	10.6	7.9	10.1	9.0	10.3	9.0	10.6
**Complications (%)**									
Local complications	29.0	28.6	30.2	29.5	28.3	**25.3**	**34.7^∗^**	29.1	28.6
Fluid collection	25.0	24.7	26.7	23.9	25.3	**22.1**	**29.8^∗^**	24.9	27.2
Pseudocyst	7.6	7.8	7.3	6.9	9.3	**6.0**	**10.6^∗^**	7.6	7.8
Necrosis	8.0	7.1	10.2	7.8	8.0	8.2	8.9	8.3	6.8
New onset diabetes	3.8	3.5	4.6	2.7	4.1	3.6	5.2	4.6	N/A
Systemic complications	7.6	**6.0**	**11.3^∗^**	**3.8**	**10.1^∗^**	6.6	9.5	7.0	10.2
Respiratory failure	4.6	**3.5**	**7.3^∗^**	**2.0**	**6.1^∗^**	4.5	4.9	4.1	7.3
Heart failure	1.8	1.4	3.0	**0.7**	**2.5^∗^**	1.9	2.0	1.9	1.5
Renal failure	2.7	**1.4**	**5.9^∗^**	**0.7**	**4.1^∗^**	**2.2**	**4.6^∗^**	2.8	2.4

*Description of the study population. Demography, etiology, and outcome of AP. Significantly different values are marked in bold digits with an asterisk. Statistical analysis is summarized in Supplementary Appendix S4.*

The major etiologies of AP were biliary stones in 37.8% of the cases of the total cohort, alcohol in 18.5%, and HL in 3.7%. OB increased the risk of biliary etiology [OR: 2.06 (CI: 1.61–2.64)]. Meanwhile, HTG-induced AP was more frequent in the presence of HL (12.9 vs. 0.1%, *p* < 0.001) compared to the non-HL group and in the presence of DM compared to the non-DM patient group [OR: 2.34 (CI: 1.39–4.00)], respectively (Table 1).

#### Obesity (Figure 1)

Obesity was less common in males [OR: 0.75 (CI: 0.58–0.95)]. There was no difference between the ages of the OB and non-OB groups (56.3 ± 15.2 vs. 55.4 ± 17.7, *p* = 0.398), although the age distribution showed a larger proportion of obese patients in the older age groups.

**FIGURE 1:**
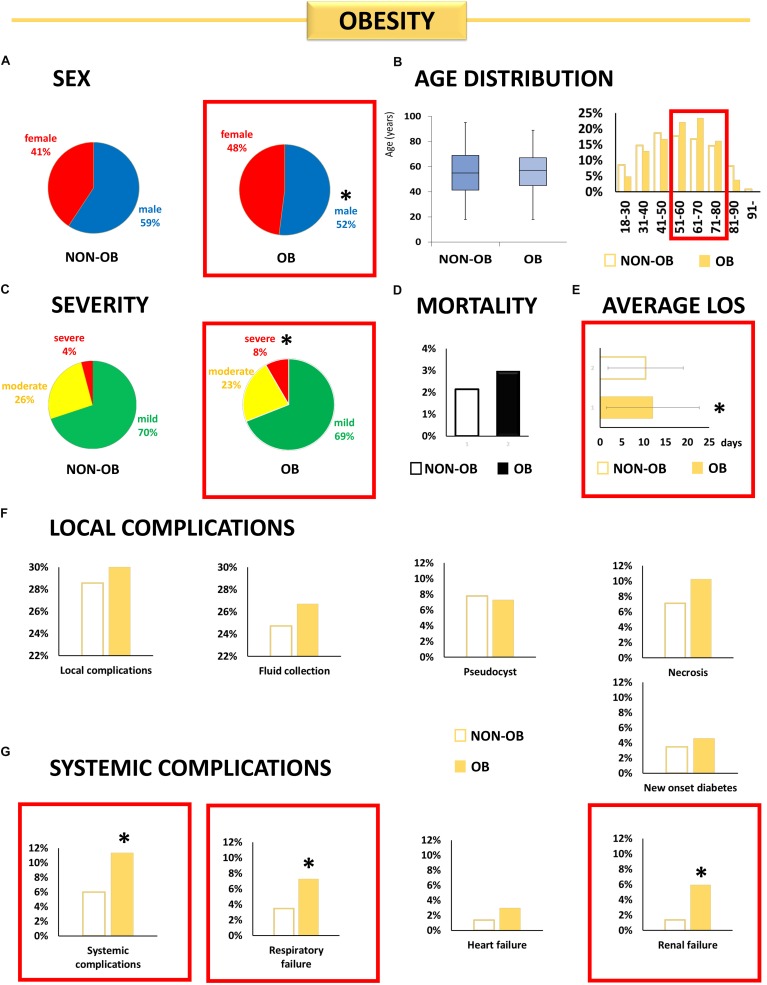
Individual effect analysis. OB and the outcome of AP. **(A)** The share of male patients was lower in the OB group [^∗^OR: 0.75 (CI: 0.58–0.95)]. **(B)** There is no difference in the average age between the OB and non-OB groups (*p* = 0.398). **(C)** Obese patients have more than double the risk of severe AP [^∗^OR: 2.15 (CI: 1.31–3.54)]. **(D)** Obese patients did not have a higher risk of mortality. **(E)** Obese patients spent more time in the hospital (^∗^*p* = 0.008). **(F)** More local complications were observed in the OB group, although the difference was not significant. **(G)** Obese patients had a higher risk of systemic complications [^∗^OR: 1.99 (CI: 1.30–3.05)], respiratory failure [^∗^OR: 2.15 (CI: 1.26–3.65)], and renal failure [^∗^OR: 4.56 (CI: 2.23–9.32)].

**FIGURE 2:**
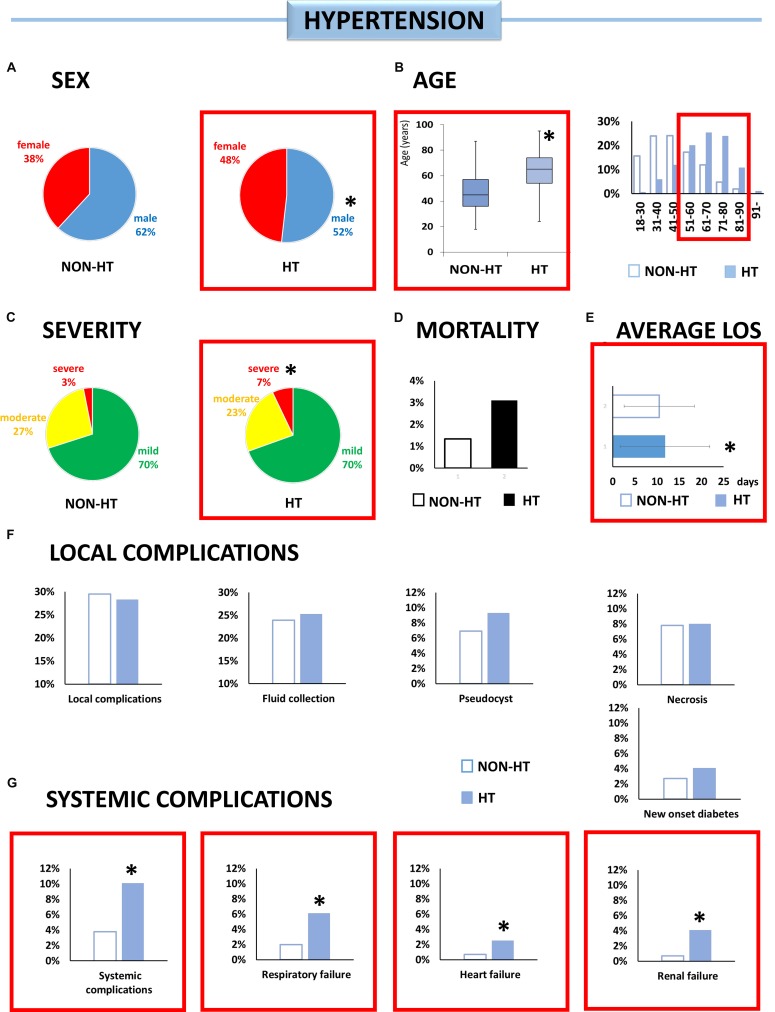
Individual effect analysis. HT and the outcome of AP. **(A)** There are fewer male patients with HT [^∗^OR: 0.66 (CI: 0.52–0.84)]. **(B)** Patients with HT are older than patients without it (^∗^*p* < 0.001). **(C)** Hypertensive patients have more than double the risk of the severe form of AP [^∗^OR: 2.39 (CI: 1.30–4.38)]. **(D)** The risk of mortality was not higher in the HT group. **(E)** Patients with HT spent more time in the hospital (^∗^*p* = 0.020). **(F)** There was a higher incidence of fluid collection, pseudocysts, and new onset diabetes, although the difference was not significant. **(G)** Hypertensive patients have a higher risk of systemic complications [^∗^OR: 2.83 (CI: 1.64–4.88)], respiratory failure [^∗^OR: 3.14 (CI: 1.51–6.52)], heart failure [^∗^OR: 3.82 CI: (1.11–13.11)], and renal failure [^∗^OR: 6.40 (CI: 1.93–21.17)].

Obesity increased the risk of severe AP [OR: 2.15 (CI: 1.31–3.54)] but showed no relation to the mortality rate [OR: 1.39 (CI: 0.66–2.96)]. OB did not influence the incidence of local complications (Figure 2F) but increased the risk of systemic complications [OR: 1.99 (CI: 1.30–3.05)], and respiratory [OR: 2.15 (CI: 1.26–3.65)] and renal [OR: 4.56 (CI: 2.23–9.32)] failure in AP. Obese patients spent a longer time in the hospital (12.1 vs. 10.4 days, *p* = 0.008) (Figure 2G).

##### Independent effect

Logistic regression revealed that OB was an independent predictive factor for renal failure [OR: 2.98 (CI: 1.33–6.66)] (Table 2).

**TABLE 2:** Independent effect of components of MetS, including age, in the logistic regression.

**MetS component**	**Outcome parameter**	**OR**	**95% CI**
	Severity	1.38	0.73–2.58
	Mortality	1.06	0.38–2.96
	Local complications	0.99	0.72–1.37
	Fluid collection	1.05	0.75–1.48
	Pseudocyst	0.85	0.50–1.44

OB	Necrosis	1.48	0.89–2.45
	New onset of diabetes	1.52	0.73–3.14
	Systemic complication	1.35	0.79–2.30
	Respiratory failure	1.52	0.77–3.02
	Heart failure	2.45	0.88–6.78
	**Renal failure**	**2.98**	**1.33–6.66**
	**Severity**	**3.41**	**1.39–8.37**
	Mortality	4.50	0.91–22.20
	Local complications	1.22	0.85–1.75
	Fluid collection	1.42	0.97–2.08
	Pseudocyst	1.55	0.85–2.81

HT	Necrosis	1.36	0.76–2.43
	New onset of diabetes	1.56	0.66–3.65
	**Systemic complication**	**2.64**	**1.27–5.51**
	Respiratory failure	1.59	0.63–4.00
	Heart failure	1.41	0.36–5.54
	**Renal failure**	**7.46**	**1.61–34.49**
	Severity	1.40	0.73–2.67
	Mortality	0.61	0.19–2.00
	**Local complications**	**1.51**	**1.10–2.07**
	Fluid collection	1.32	0.94–1.84
	Pseudocyst	1.58	0.95–2.61

HL	Necrosis	1.06	0.63–1.78
	**New onset of diabetes**	**2.55**	**1.26–5.19**
	Systemic complication	1.34	0.77–2.32
	Respiratory failure	0.90	0.43–1.90
	Heart failure	1.59	0.54–4.67
	Renal failure	1.93	0.85–4.38
	Severity	0.48	0.20–1.16
	Mortality	0.46	0.10–2.14
	Local complications	0.84	0.56–1.28
	Fluid collection	1.02	0.67–1.56
	Pseudocyst	1.01	0.53–1.91

DM	Necrosis	0.53	0.24–1.14
	New onset of diabetes	N/A	N/A
	Systemic complication	0.92	0.48–1.74
	Respiratory failure	1.48	0.68–3.20
	Heart failure	0.32	0.07–1.53
	Renal failure	0.43	0.15–1.22

*OB is an independent predictive factor for renal failure; HT for severity; and systemic complications, renal failure, and hyperlipidemia for local complications and for a new diagnosis of diabetes mellitus. OR, odds ratio; CI, confidence interval. Statistically significant values (ORs with CIs) are marked in bold digits.*

#### Hypertension (Figure 2)

Patients with HT were 17.6 years older on average (63.8 ± 14.1 vs. 46.2 ± 15.2, *p* < 0.001). Male gender was associated with a lower risk of HT [OR: 0.66 (CI: 0.52–0.84)].

**FIGURE 3:**
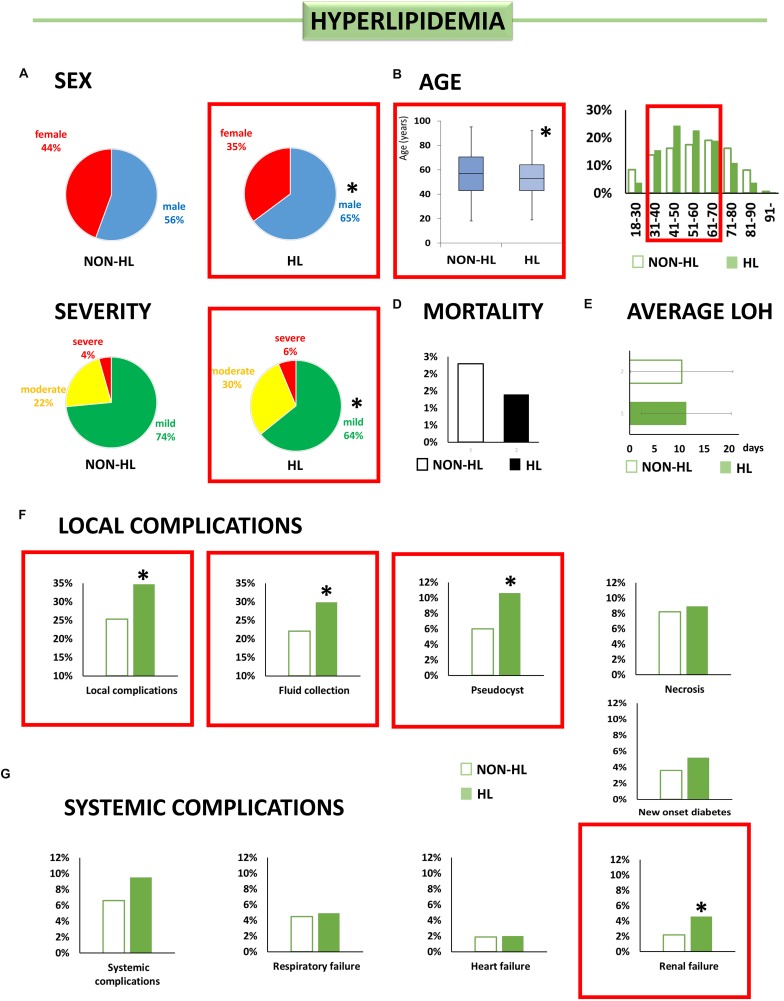
Individual effect analysis. HL and the outcome of AP. **(A)** There are more male patients with HL [^∗^OR: 1.47 (CI: 1.12–1.92)]. **(B)** Patients with HL are younger than patients without it (^∗^*p* < 0.001). **(C)** Hyperlipidemic patients have a lower chance of having mild AP [^∗^OR: 0.65 (CI: 0.49–0.85)]. **(D)** Patients with HL did not have a higher risk of mortality. **(E)** Patients with HL spent more time in the hospital (^∗^*p* = 0.053). **(F)** HL increases the risk of local complications [^∗^OR: 1.55 (CI: 1.17–2.05)], acute fluid collection [^∗^OR 1.48 (CI: 1.11–1.99)], and pseudocysts [^∗^OR 1.81 (CI: 1.14–2.88)]. **(G)** Hyperlipidemic patients have a higher risk of renal failure [^∗^OR 2.17 (CI: 1.51–4.43)].

Hypertension increased the risks of severe AP [OR: 2.39 (CI: 1.30–4.38)], systemic complications [OR: 2.83 (CI: 1.64–4.88)], and respiratory [OR: 3.14 (CI: 1.51–6.52)], heart [OR: 3.82 (CI: 1.11–13.11)], and renal failure [OR: 6.40 (CI: 1.93–21.17)]. HT was also associated with longer hospitalization (11.8 vs. 10.5 days, *p* = 0.020) (Figure 3E).

##### Independent effect

Logistic regression revealed that HT was a predictive factor for severity [OR: 3.41 (CI: 1.39–8.37)], systemic complications [OR: 2.64 (CI: 1.27–5.51)], and renal failure [OR: 7.46 (CI: 1.61–34.49)] as well (Table 2).

#### Hyperlipidemia (Figure 3)

Contrary to OB and HT, HL was associated with younger age (54.0 ± 14.5 vs. 56.4 ± 17.8, *p* = 0.032) and a higher rate among male patients [OR: 1.47 (CI: 1.12–1.92)].

For patients with HL, the chance of having mild AP was lower [OR: 0.64 (CI: 0.49–0.85)], but HL had no significant effect on mortality. HL increased the risk of local complications [OR: 1.55 (CI: 1.17–2.05)], and, within local complications, acute fluid collections and pseudocyst formation were more frequent [OR: 1.48 (CI: 1.11–1.99); OR: 1.81 (CI: 1.14–2.88), respectively]. HL also increased the risk of renal failure [OR: 2.17 (CI: 1.06–4.43)].

##### Independent effect

Logistic regression revealed that HL was an independent predictive factor for local complications [OR: 1.51 (CI: 1.10–2.07)] and for a new diagnosis of DM [OR: 2.55 (CI: 1.26–5.19)] (Table 2).

#### Diabetes Mellitus (Figure 4)

Patients with DM were older (61.7 ± 13.9 vs. 54.5 ± 17.3, *p* < 0.001), while there was no difference in the gender ratio between the DM and non-DM groups [OR: 1.19 (CI: 0.88–1.62)] (Supplementary Appendix S4). Statistical analyses demonstrated no significant relation between DM and the severity, mortality, and complications of AP.

**FIGURE 4:**
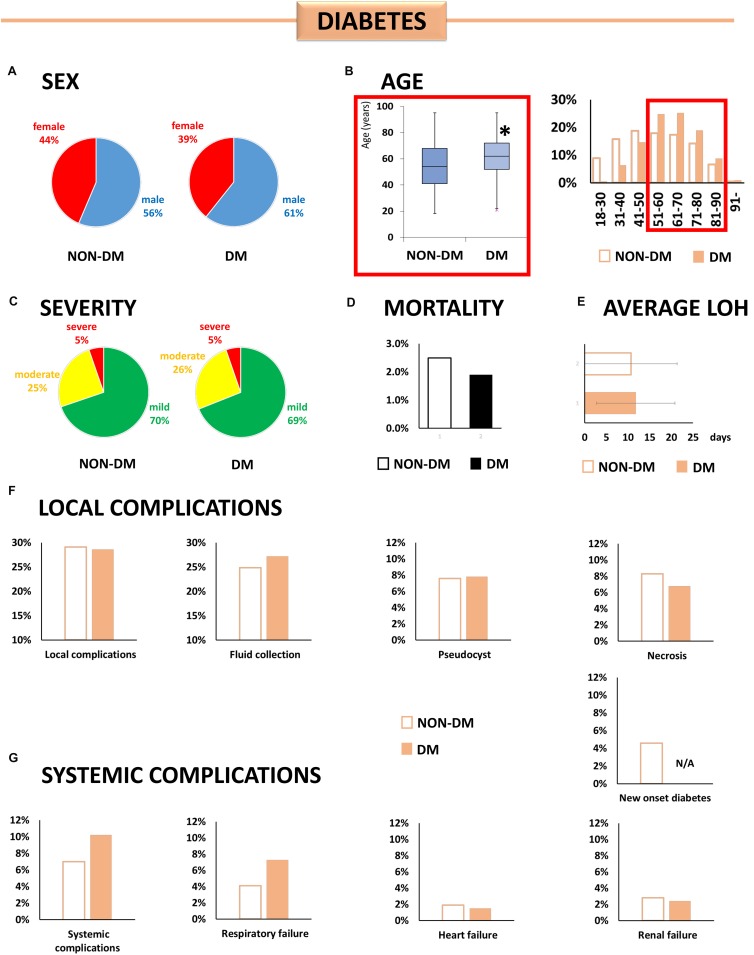
Individual effect analysis. DM and the outcome of AP. **(A)** There is no significant difference in sex between the two groups. **(B)** Patients with diabetes are older than patients without it (^∗^*p* < 0.001). **(C,D)** Diabetic patients did not have a higher risk of moderately severe or severe AP or mortality in our cohort. **(E)** There is no difference in LOS between the two groups (*p* = 0.139). **(F,G)** As regards local or systemic complications, there are no differences between diabetic and non-diabetic patients in our cohort.

### Joint Effect Analysis

**FIGURE 5:**
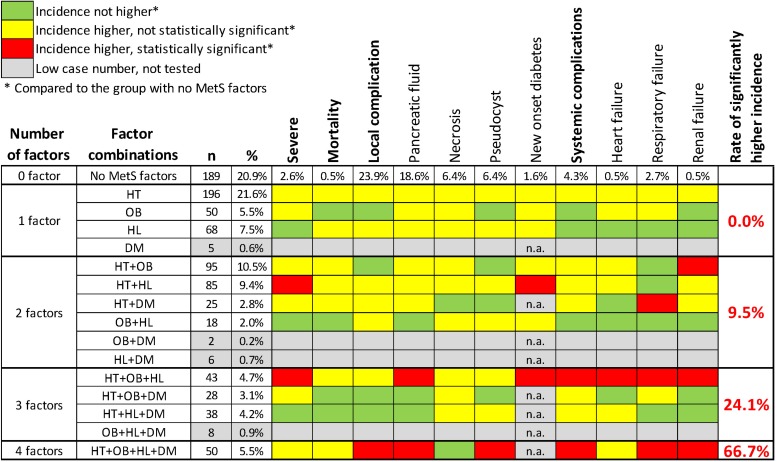
Joint effect analysis. The effect of MetS factor combinations on the outcome of AP. The more MetS factors are present, the more significantly higher incidence of the different outcome parameters can be observed. Statistical analysis is summarized in Supplementary Appendix S5.

A total of 906 patients in our cohort (mean age ± *SD*: 56.9 ± 16.7 years, males vs. females: 57.3 vs. 42.7%) were eligible for the “joint effect analysis.” 189 patients (20.9%) had no components of MetS, 294 (32.5%) had OB, 560 (61.8%) had HT, 316 (34.9%) had HL, and 162 (17.9%) had DM. We formed groups of patients according to the factor combinations they had and compared the outcome parameters between the different factor combinations and the group of no MetS factors one by one (Supplementary Appendix S5). The presence of two, three, or four MetS factors significantly increased the rate of worse outcome parameters by 9.5, 24.1, and 66.7%, respectively (Figure 5).

## Discussion

### Summary of Findings

Our results demonstrated in a large database of prospectively collected cases that the components of MetS deteriorate the outcome of AP. OB was shown to be an independent risk factor for renal failure and was associated with a longer hospital stay. HT was proved to be an independent risk factor for severity of AP and increased the risk of renal failure, while patients with HT spent a longer time in hospital. HL increased the risk of local complications, renal failure, and the new diagnosis of DM. Preexisting DM did not change the outcome of AP. Our study demonstrated that the more components of MetS the patients had, the higher the rate of worse outcome parameters was observed.

The incidence of AP is increasing, and this is partly due to the rising prevalence of OB, which stimulates gallstone formation and increases HL, both causing AP (Yadav and Lowenfels, 2013; Bonfrate et al., 2014). Indeed, biliary AP was more frequent in obese patients compared to the total cohort in our study.

To date, several cohort studies and a systematic review have reported that OB increases the severity, mortality, and occurrence of local and systemic complications in AP. However, these results are conflicting on the link between OB and outcomes in AP (Dobszai et al., 2019). The reason behind this conflict may be that most of the included studies reported unadjusted analysis; therefore, it cannot be clarified whether OB is an independent prognostic factor in AP or not (Dobszai et al., 2019). In a recent individual patient data meta-analysis, where confounders were adjusted, OB was independently associated with the development of organ failure and multiple organ failure in AP; however, there was no relation between OB and mortality, necrosis, and intervention (Premkumar et al., 2015). These data are in agreement with our results, where OB was demonstrated to be an independent predictive factor for renal failure but did not modify the mortality rate (Table 2).

A possible mechanism by which OB is associated with a higher risk of renal failure is lipotoxicity.

Obesity is associated with elevated levels of intrapancreatic fat and with elevated visceral fat surrounding the pancreas (Smeets et al., 2019). This hypothesis is also supported by experimental data. A long-term high-fat diet caused acinar cell injury and pancreatic fibrosis via fat accumulation in pancreatic acinar cells (Matsuda et al., 2014). It has also been suggested that intrapancreatic fat, which may cause metabolic and inflammatory processes, is associated with OB (Majumder et al., 2017). In addition, in the presence of intrapancreatic fat, pancreatic lipases are released in AP digest adipocytes, resulting in an outflow of unsaturated fatty acids into the circulation; they are toxic and can act as proinflammatory mediators and are implicated in the development of systemic inflammation and organ failure (Navina et al., 2011).

Hypertension was independently associated with the severity of AP and the rate of renal failure in our study. To the best of our knowledge, no study has ever analyzed the effect of arterial HT on the outcome of AP. The underlying mechanisms by which HT deteriorates the outcome of AP is unclear. It has been suggested that the sympathetic nervous system may act as an amplifier of the blood pressure elevation and may be involved in the development of HT-related complications. Sympathetic activation favors the development and progression of vascular hypertrophy and remodeling and contributes to impairing arterial distensibility and vascular compliance (Seravalle et al., 2014). The presence of a hyperadrenergic state and microvascular and macrovascular structural changes in the arteries may be responsible for the deteriorative effects of HT (Smits and van Geenen, 2011).

Preexisting HL was shown to be independently associated with local complications and renal failure in our study. Our results are in line with those of a recent meta-analysis, which reported that the presence of HTG significantly elevated the risk of renal failure but did not increase the risk of mortality in AP (Kiss et al., 2018). However, HTG also significantly elevated the risk of severe AP in this meta-analysis (Kiss et al., 2018), while HL did not increase the risk of severe AP in our study. This discrepancy can be explained by the fact that (1) most of the studies included in the meta-analysis reported an unadjusted analysis, and, therefore, the independent effect of HTG in AP cannot be elucidated; and (2) the HL group in our study included patients with either hypercholesterinemia and/or HTG, while patients with HTG only were included in the meta-analysis. One possible mechanism by which HL increases local and systemic complications in AP is the formation and toxic effect of unsaturated fatty acid by pancreatic lipases. In addition, in the case of HTG, the chylomicron concentration is elevated. As a result, blood viscosity increases, thus impairing blood flow and causing pancreatic ischemia and acidosis (Pedersen et al., 2016).

There is a special relationship between the exocrine and endocrine pancreas. Experimental data suggest that insulin has a local protective effect on acinar cells during pancreatitis. Pancreatitis evoked by L-arginine causes severe acinar cell necrosis in most of the territory of the exocrine pancreas. However, acinar cells located around the islets of Langerhans remain totally intact (Hegyi et al., 1997). In addition, we also confirmed that if the beta cells are destroyed by streptozotocin treatment prior to the induction of AP, this locally visible protective effect disappears irrespectively of exogenous insulin administration (Takacs et al., 2001). Unfortunately, in our registry analysis, we could not investigate the local effects of insulin. Here we showed that preexisting DM does not significantly influence severity, mortality, or rate of complications in AP in our cohort. We hypothesized that our cohort was not sufficiently large to determine a significant difference. We have recently published a meta-analysis in which DM significantly elevated both local and systemic complications when an analysis was conducted of 354,880 cases (Miko et al., 2018). However, it is clearly impossible to collect this number of patients in a single cohort. Furthermore, intensive care unit mortality only grew significantly with higher mean blood glucose concentration in non-DM patients but not in DM patients (Egi et al., 2008; Pedersen et al., 2016). In agreement with our results, critically ill patients with DM did not have higher mortality compared to non-DM patients (Whitcomb et al., 2005).

Older age was demonstrated to be independently associated with pulmonary and heart failure in our study (Table 2B). Older age has been investigated extensively as a marker of severity and mortality in AP and is included in the APACHE II score, Ranson score, Bedside Index of Severity in AP (BISAP) score, and Japanese Severity Score (JSS) as a marker of severity (Graham et al., 2010). However, after adjusting for comorbid disease, only the very extreme age (>85 years old) was associated with 30-day in-patient mortality and persistent organ failure in a recent prospective, multicenter study (Mounzer et al., 2012). Our results are in line with a recent cohort analysis that found that elderly patients had a significantly higher risk of developing systemic complications, while high mortality in this group is due to the effect of severe comorbidities (Szakacs et al., 2018).

**TABLE 3:** Logistic regression.

	Severity	1.01	0.99–1.03
	Mortality	1.02	0.98–1.05
	Local complications	0.99	0.98–1.00
	Fluid collection	0.99	0.98–1.00
	Pseudocyst	1.00	0.98–1.01
Age	Necrosis	0.99	0.97–1.00
	New onset of diabetes	1.01	0.99–1.04
	Systemic complication	1.01	0.99–1.03
	**Respiratory failure**	**1.03**	**1.01–1.06**
	**Heart failure**	**1.05**	**1.01–1.09**
	Renal failure	1.00	0.97–1.03

*Older age was demonstrated to be independently associated with respiratory and heart failure in our study. Statistically significant values (ORs with CIs) are marked in bold digits.*

Patients with AP often develop diabetes during and after the attack of AP (Moran et al., 2018); however, the risk of DM was not fully evaluated. The severity of AP, its etiology, and individuals’ age and sex had a minimal effect on the development of newly diagnosed diabetes in AP (Moran et al., 2018). We showed that HL is an independent risk factor for the development of newly diagnosed DM in AP. High cholesterol and triglyceride levels increase the risk of DM, a finding supported by earlier studies (von Eckardstein and Sibler, 2011; Das et al., 2014). We can hypothesize that the predisposition to DM caused by dyslipidemia was manifested during AP. This finding emphasizes the need for a thorough screening for DM in AP patients with HL. Moreover, all AP patients should be followed and screened for DM as hyperglycemia stimulates the proliferation of pancreatic stellate cells and collagen secretion, while hypoinsulinemia inhibits acinar cell growth and synthesis of pancreatic enzymes and therefore facilitates fibrosis of the pancreas and might cause chronic pancreatitis (Czako et al., 2009).

### Strengths and Limitations

The main strength of the present study is that it has a large sample size of prospectively collected cases from hospitals in multiple countries, including tertiary and non-tertiary centers. Furthermore, a logistic regression analysis was applied to control confounding variables, and the independent prognostic factors of the components of MetS were analyzed for AP. Finally, our study is the first to report the relation between the outcome of AP and the presence of arterial HT and to analyze the influence of the combined presence of the components of MetS on the outcome of AP.

The present study has limitations. First, since APR is a multicenter prospective registry and not an observational trial, our findings are affected by confounding factors or selection bias. Second, our study design is cross-sectional, thus precluding any causal interferences about the directionality of the relations observed in our study; therefore, long-term clinical outcomes could not be evaluated. Accordingly, long-term prospective trials are needed in the future. Third, our study assessed the effect of HL, not HTG, thus not fully suiting the definition of MetS. Fourth, peripancreatic fluid accumulations could not always be adequately defined according to the modified Atlanta classification. Acute fluid collection and acute necrotic fluid collection, pseudocysts, and walled-off pancreatic necrosis could not always be differentiated because abdominal CT was not performed in all cases. Therefore, peripancreatic fluid collections without a definitive wall were named as acute fluid collections and with a wall as pseudocysts.

## Conclusion

In conclusion, the components of MetS deteriorate the outcome of AP. OB, HT, and HL are independent risk factors for a number of complications. HT is an independent risk factor for severity as well. The more elements of MetS are present, the higher the risk for complications. It is important to search for and follow up on the components of MetS in AP.

## Data Availability

All datasets generated for this study are included in the manuscript and/or the Supplementary Files.

## Ethics Statement

The study protocol was approved by the Scientific and Research Ethics Committee of the Medical Research Council (22254-1/2012/EKU). All patients provided written informed consent to participate in the study.

## Author Contributions

AS, AP, and PH contributed to the design of the research. AP, ÁV, JB, SG, PS, FI, AH, IT, HF, MP, MV, JH, JN, AM, EM, VS, LC, and TT collected the data. AP, KM, DD, DI, and BK assessed the data quality. NG and AS processed the data and conducted the analysis. AS and PH designed the figures. AS, LC, and BE drafted the manuscript. PH supervised and coordinated the work. All the authors discussed the results and commented on the manuscript.

## Conflict of Interest Statement

The authors declare that the research was conducted in the absence of any commercial or financial relationships that could be construed as a potential conflict of interest.

## Supplementary Material

APPENDIX S1 Center distribution.

APPENDIX S2 Demography and representativeness of study populations.

APPENDIX S3 Data quality.

APPENDIX S4 Statistics of individual effect analysis.

APPENDIX S5 Joint effect analysis. **(A)** Description of demography and incidences of the different outcomes. **(B)** Statistics.

APPENDIX S6 Database of the analysis.

## Abbreviations

AP: acute pancreatitis
APR: Acute Pancreatitis Registry
BMI: body mass index
CI: 95% confidence interval
DM: diabetes mellitus
HL: hyperlipidemia
HPSG: Hungarian Pancreatic Study Group
HT: hypertension
LOS: length of hospital stay
MetS: metabolic syndrome
OB: obesity
OR: odds ratio
SD: standard deviation.
