# Supplementary figures and images for: Multiple Hits in Acute Pancreatitis: Components of Metabolic Syndrome Synergize Each Other’s Deteriorating Effects
Source: Front Physiol. 2019 Sep 20;10:1202. doi: 10.3389/fphys.2019.01202 (PMC6763590; doi:10.3389/fphys.2019.01202)

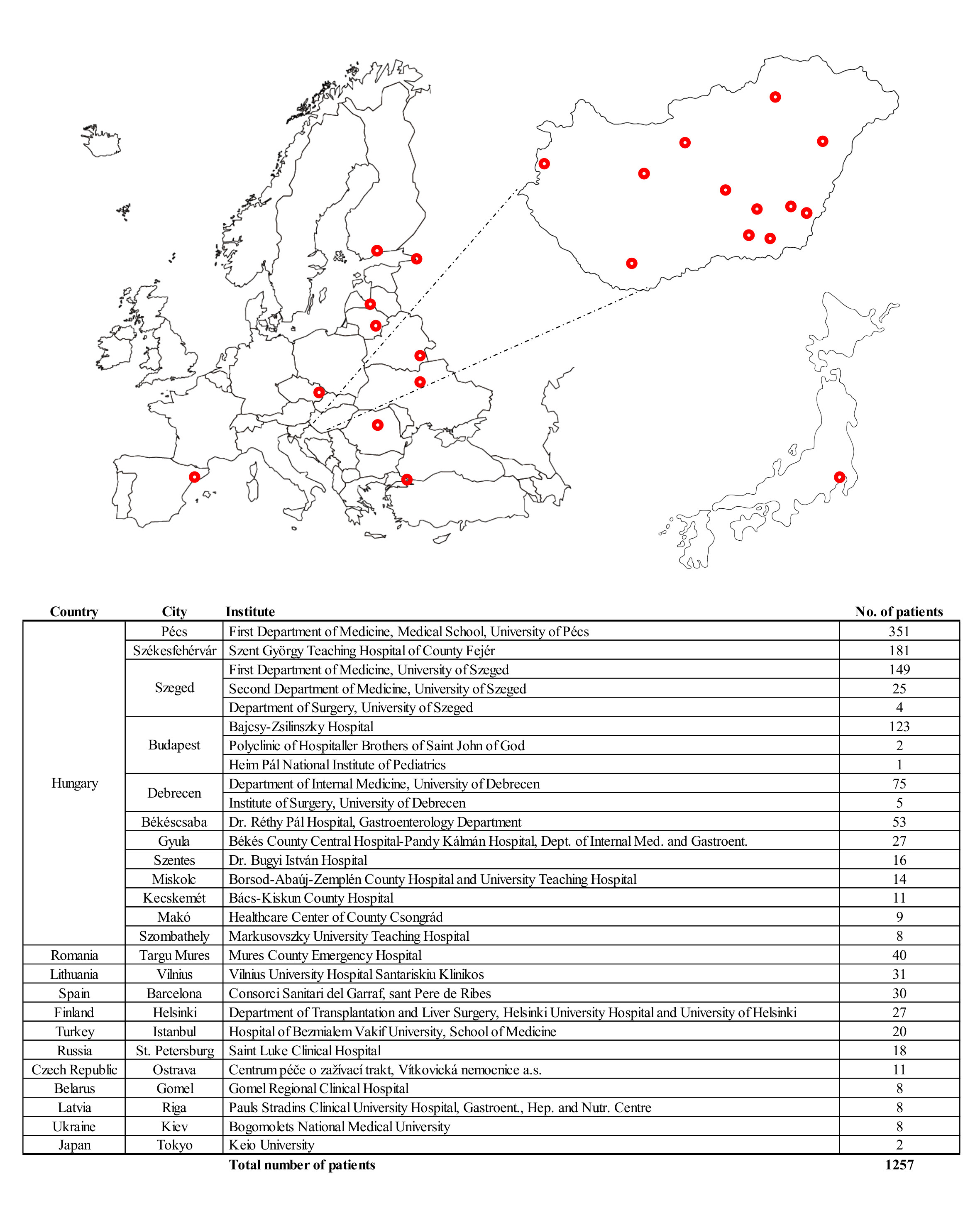

Supplement: APPENDIX S1 — Center distribution. [file Image_1.JPEG]

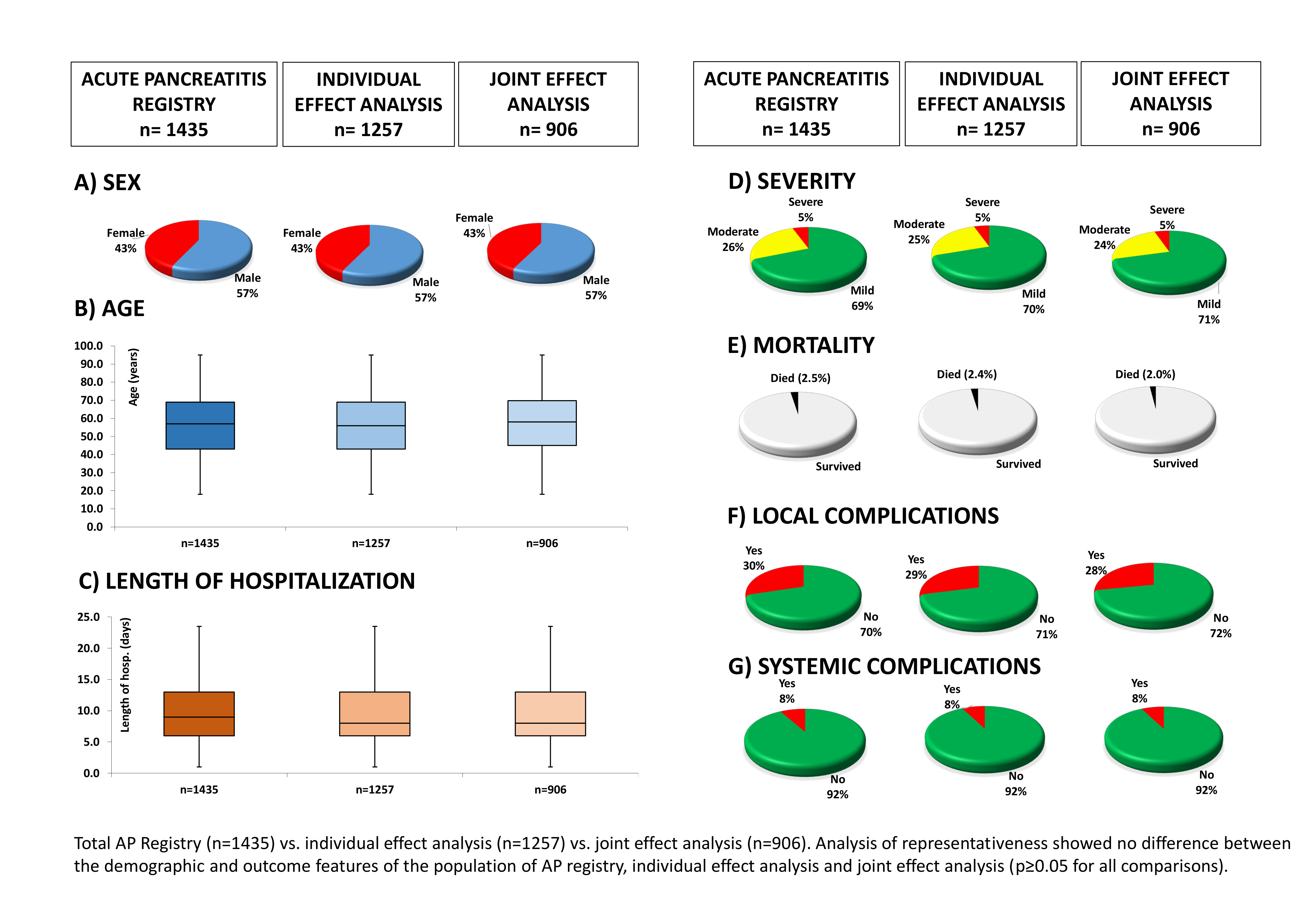

Supplement: APPENDIX S2 — Demography and representativeness of study populations. [file Image_2.JPEG]

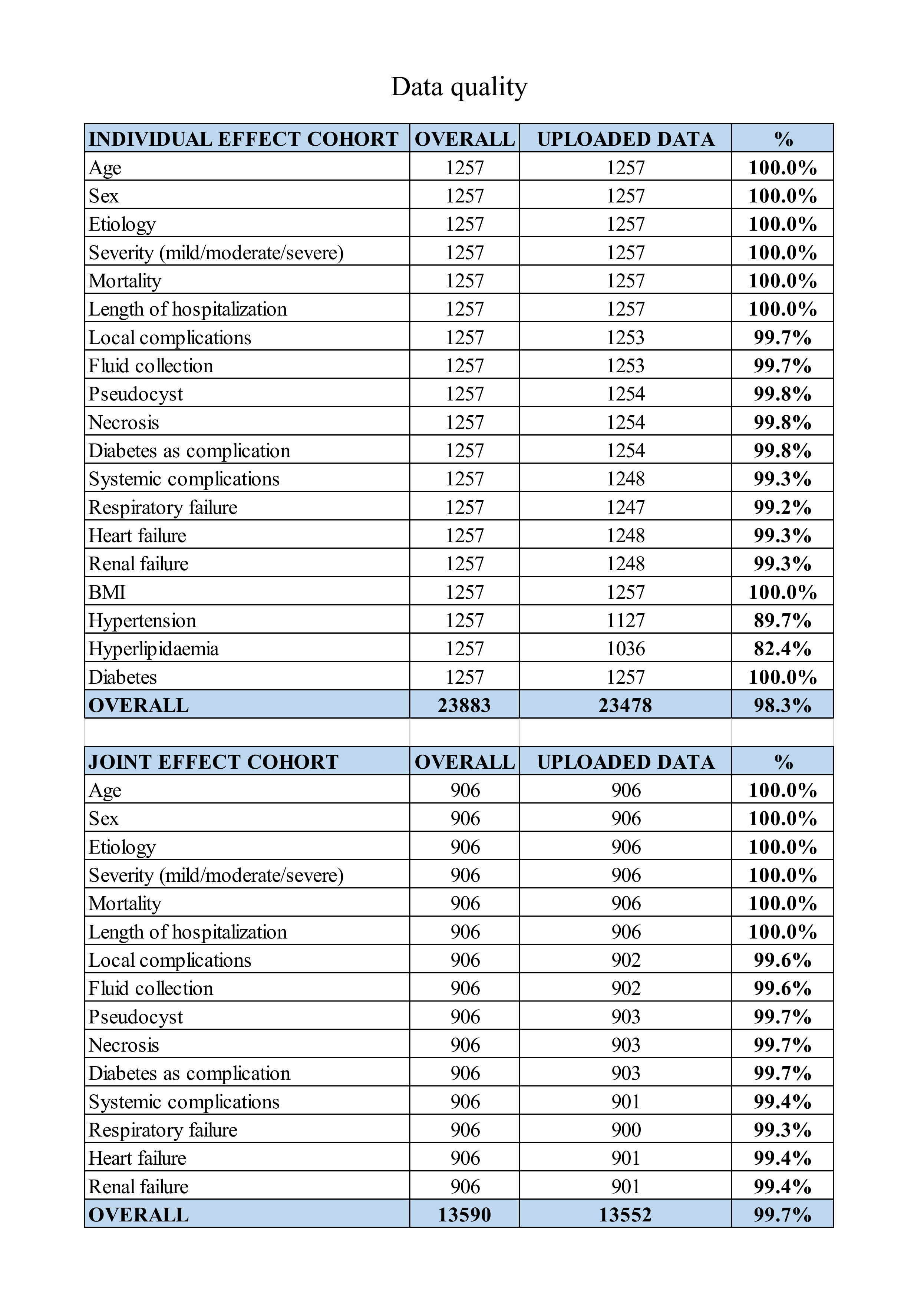

Supplement: APPENDIX S3 — Data quality. [file Image_3.JPEG]
